# Floating Chip Mounting System Driven by Repulsive Force of Permanent Magnets for Multiple On-Site SPR Immunoassay Measurements

**DOI:** 10.3390/s121013964

**Published:** 2012-10-17

**Authors:** Tsutomu Horiuchi, Tatsuya Tobita, Toru Miura, Yuzuru Iwasaki, Michiko Seyama, Suzuyo Inoue, Jun-ichi Takahashi, Tsuneyuki Haga, Emi Tamechika

**Affiliations:** 1 NTT Microsystem Integration Laboratories, Nippon Telegraph and Telephone Corporation (NTT), Morinosato-Wakamiya, Atsugi-shi, Kanagawa 2430198, Japan; E-Mails: miura.toru@lab.ntt.co.jp (T.M.); iwasaki.yuzuru@lab.ntt.co.jp (Y.I.); seyama.michiko@lab.ntt.co.jp (M.S.); inoue.suzuyo@lab.ntt.co.jp (S.I.); takahashi.junichi@lab.ntt.co.jp (J.T.); haga.tsuneyuki@lab.ntt.co.jp (T.H.); tamechika.emi@lab.ntt.co.jp (E.T.); 2 NTT Advanced Technology, Morinosato-Wakamiya, Atsugi-shi, Kanagawa 2430198, Japan; E-Mail: tatsuya.tobita@ntt-at.co.jp

**Keywords:** SPR, on-site, portable, immunoassay, magnet

## Abstract

We have developed a measurement chip installation/removal mechanism for a surface plasmon resonance (SPR) immunoassay analysis instrument designed for frequent testing, which requires a rapid and easy technique for changing chips. The key components of the mechanism are refractive index matching gel coated on the rear of the SPR chip and a float that presses the chip down. The refractive index matching gel made it possible to optically couple the chip and the prism of the SPR instrument easily via elastic deformation with no air bubbles. The float has an autonomous attitude control function that keeps the chip parallel in relation to the SPR instrument by employing the repulsive force of permanent magnets between the float and a float guide located in the SPR instrument. This function is realized by balancing the upward elastic force of the gel and the downward force of the float, which experiences a leveling force from the float guide. This system makes it possible to start an SPR measurement immediately after chip installation and to remove the chip immediately after the measurement with a simple and easy method that does not require any fine adjustment. Our sensor chip, which we installed using this mounting system, successfully performed an immunoassay measurement on a model antigen (spiked human-IgG) in a model real sample (non-homogenized milk) that included many kinds of interfering foreign substances without any sample pre-treatment. The ease of the chip installation/removal operation and simple measurement procedure are suitable for frequent on-site agricultural, environmental and medical testing.

## 1. Introduction

Immunoassay is typically used in clinical testing for evaluating the state of an illness [1–4], for measuring toxic substances in the environment [5–8], and in food inspection for the prevention of food poisoning [9]. The surface plasmon resonance (SPR) method was developed for immunoassay analysis, and is now in practical use [10–19]. This is because SPR has versatility and a fast detection ability that originates from the non-label detection principle. An SPR instrument is considered to be a potential platform for on-site measurement and point-of-care testing.

On-site measurement requires a portable instrument and a conventional flow system for the sample liquid. A micro-pump system driven by capillary force is the simplest approach and has been studied for this purpose and for many other applications [20–25]. We have developed an on-site SPR immunoassay system consisting of a portable SPR instrument and a single-use microfluidic immunoassay chip that includes capillary pumps [26]. The microfluidic device could perform the immunoassay analysis of a real liquid sample via direct injection of the sample into the inlet hole using a pipette and without any pretreatment. This flow analysis requires no external mechanical pump, tube connection, or cleaning operation. A single measurement takes several minutes. This easy and simple protocol is particularly advantageous for performing on-site immunoassays.

Although the measurement takes only a few minutes, a further several minutes are required before starting the next measurement because the chip replacement operation is time-consuming. An SPR measurement is a precise optical measurement and an exact chip arrangement is needed to achieve optical coupling between the chip surface and the cylindrical prism of the SPR instrument. Refractive index matching liquid is usually used for this purpose. However this material is sticky, and so removing and cleaning the prism are time-consuming operations. In actual use, even in the field, a sufficient quantity of cleaning material (cleaning liquid, wiping paper) must be provided. Consequently, there are problems related to cost and waste management.

Some commercially available large scale and non-portable SPR instruments use an easy-to-operate chip mounting system. However, a refractive index matching gel-like material regarded as a maintenance component has to be employed on the prism surface. In a portable SPR system for on-site use, small dirt particles may penetrate between the prism and the chips. An open structure or maintenance free chip mounting system is required. In addition, to give priority to portability, we must reduce the need for large accessories for the highly accurate mounting mechanism or temperature regulation system. A small simple mounting system is required for use in the field.

Some alternatives to refractive index matching liquid and gels have been studied for conventional optical coupling. They are plasticized poly(vinyl chloride) (PVC) film [27] and poly (dimethylsiloxane) (PDMS) film [28]. However the PVC film had the same drawbacks as the matching oils and gels. On the other hand, the PDMS film can be stripped and removed very easily, however there is a large difference between the refractive indexes of PDMS (1.41) and the commonly-used optical glass BK7 (1.51). This refractive index mismatch makes the dip in the SPR profile curve (relative reflectance as a function of incident angle) broader and shallower, and the local minimum position changes. These changes in the SPR profile curve are not favorable with regard to the reliability and the signal to noise ratio when measuring trace analyte concentrations. The possibility of the CCD array becoming out of range as a result of the minimum shift of the SPR profile curve is also unacceptable. Another disadvantage of using these refractive index films is the high probability of accidental air bubble penetration.

Recently, many kinds of silicone gels have been developed and widely applied in many industrial fields. Some of these are non-sticky and easily removable silicone gels with optimum refractive indexes as optical coupling materials. If refractive index matching gel is used as an optical coupling material between a chip and a prism, a highly accurate mechanism for positioning the chip is required when mounting it on the SPR instrument. The SPR profile curve is greatly affected by the thickness of the refractive index matching gel and the tilt of the chip against the prism. The chip must be kept in a parallel position at a constant distance to the prism if we are to obtain a reasonable result that can be compared with that of other chips.

One simple method involves applying downward force against the elastic force of the gel. Mechanical components such as screws, springs weights or magnets have been considered for this purpose. We thought the use of magnetic force to be a practical and reasonable method for simple and easy tool-free chip replacement. The use of magnetic force has been studied in relation to reversible sealing components such as tube connectors and clamps for bonded-type fluidic chips, to allow microfluidic experiments to be set up simply and easily [29,30]. The magnetic force used in these applications was the attractive force, which shows the tightest sealing when the distance between the magnet and the other component is shortest. Although this is reasonable for protecting against leaks, a strong force must be applied or a special mechanism is required to remove the tube connectors or clamps when breaking the seal.

In this work, we have developed a low cost single-use immunoassay chip that includes optical coupling gel sheets and its installation/removal mechanism. This mechanism is driven by the repulsive force of permanent magnets and allows us to change chips rapidly, thus satisfying the demand for a large number of on-site SPR measurements. We have demonstrated the immunoassay of a spiked model antigen (human IgG) in a model real sample using our sensor chip and its mounting system. The model real sample was non-homogenized milk that naturally included many interfering foreign substances but none of the model antigen, human-IgG. We confirmed the usefulness of this chip mounting system.

## 2. Experimental

### 2.1. Instrument, Materials and Reagents

**Figure 1.**
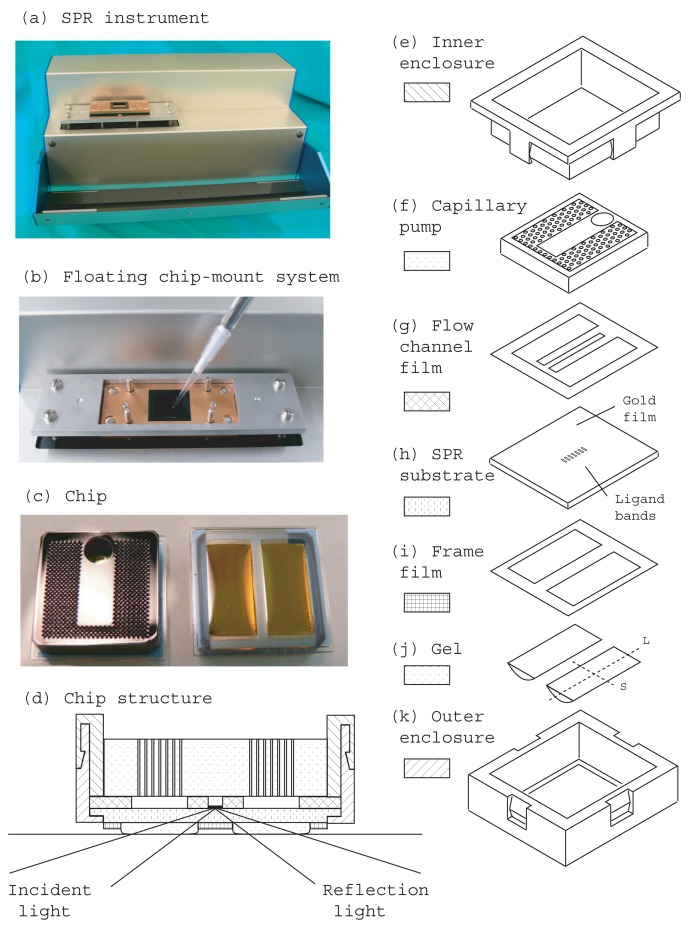
Portable SPR instrument (**a**) and floating chip mounting system (**b**). Images of main measurement chip components, top view (**c**, left) and rear view (**c**, right). Cross sectional view of chip (**d**) and its components (**e**–**k**). Hatched areas in (d) correspond to the legends for components (e-k).

**Figure 2.**
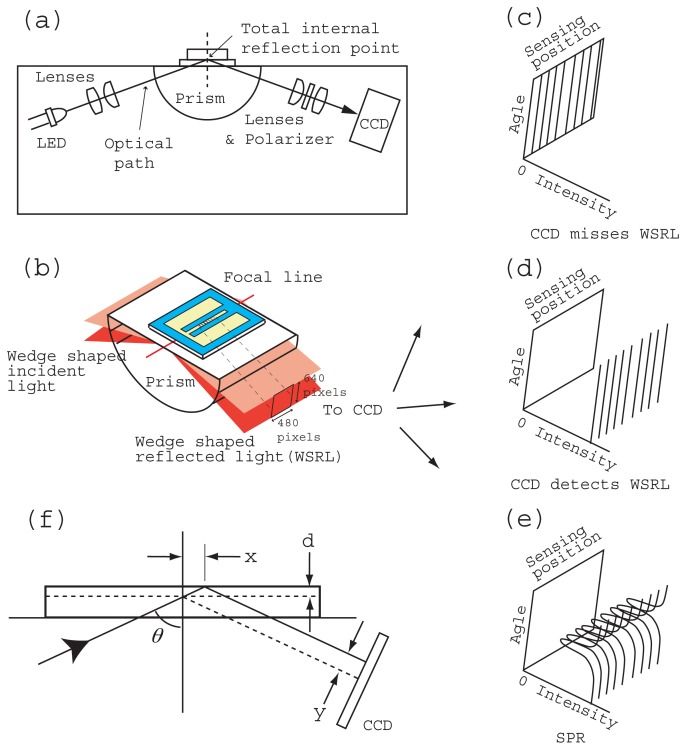
Schematic diagram of the optical configuration of an SPR instrument (**a**). Optical configuration at the interface of the prism and the sensor chip (**b**). The CCD cannot detect wedge shaped reflected light when the sensor chip is mounted incorrectly (**c**). The CCD can detect all wedge shaped reflected light when the sensor chip is mounted correctly (**d**). SPR curves are obtained if the sample has a resonance angle within wedge shaped light and condition (d) is satisfied (**e**). The total internal reflection point is displaced by x and the illuminated position on the CCD is displaced by y as a result of applying refractive index matching gel of thickness d (**f**).

A portable SPR instrument (290 mm (W) × 160 mm (D) × 120 mm (H)), (Smart SPR SS-1001, NTT Advanced Technology, Japan) (Figure 1(a)) and a homemade control and data acquisition program coded with LabVIEW (National Instrument, Japan) were used for the measurements. The SPR instrument had a Kretschmann type optical configuration (Figure 2(a)). The sensing area (4.5 mm × 0.3 mm) is located on the center of the focused line of a cylindrical prism (BK7, refractive index 1.51). The incident light was emitted from an LED at a wavelength of 770 nm. A CCD (480 × 640 pixels) camera detected the reflection intensity with a resolution of 4.5 mm/480 pixels in the longitudinal direction of the sensing area (Figure 2(b)). The resolution of the reflection angle was 10 degrees/640 pixels. In this instrument several threaded screw holes were prepared in advance for the user. Our mounting system used these holes and we did not modify the instrument.

The CCD output was transferred to a laptop PC (ThinkPad T7500, Lenovo Japan, Japan) through a gigabit Ethernet cable. We obtained the reflection intensity minimum SPR angle by employing a conventional centroid curve fitting program.

Black acrylic resin plates were purchased from Sumitomo Chemical Co. Ltd., (Japan). Double-sided adhesive film (TL470S 0.075 mm thickness) and single-sided adhesive film (GD-60-21S, 0.080 mm thickness) were supplied by Lintec Corporation (Japan) and Toyo Adtec Co. Ltd. (Japan), respectively.

The inner enclosure (Figure 1(e)) and outer enclosure (Figure 1(k)) were injection-molded components made of methyl methacrylate-styrene resin. The SPR substrate (Figure 1(h) 16 mm × 16 mm × 1 mm) was an injected-molded component made of amorphous cycloolefin copolymer (refractive index 1.54), APEL™. All the injection-molded components were purchased from Arkhe Co., Ltd. (Japan). Silicone gel IVS5022 (refractive index 1.51) was obtained from Momentive Performance Materials Inc. (Japan). We used a glass (BK7, refractive index 1.51) substrate (Taise, Japan) and refractive index matching oil, nD 1.5180 ± 0.0002 (Cargille-Sacher Laboratories Inc., USA) for a comparison experiment.

Neodymium magnets with yoke, NCB20 (20 mm × 13.5 mm × 5 mm, 4300 gauss surface inductive flux, 6 kg adhesive force) were obtained from Seiko Sangyo Co., Ltd. (Japan). Compression coiled springs C05-01 (0.0582 kgf/mm spring constant) were obtained from Shinsei Hatsujho Co., Ltd. (Japan).

The float (64 mm × 28 mm × 8 mm, 70.0 g weight) was composed of two custom-made copper plates, a single acrylic resin plate and two neodymium magnets as described above. The float had a square hole in its center to allow access to the chip. The float guide (126 mm × 36 mm × 4 mm, 44.8 g weight) was composed of a custom-made aluminum plate and two neodymium magnets as described above. The float guide had a rectangular hole for the float to pass through. These four magnets were attached with screws by aligning the orientation to generate repulsive forces when the float was inside the float guide hole. A 1.4 mm thick steel plate cold commercial (SPCC) quality, ferromagnetic plate was used for a comparison experiment.

A blocking reagent (Block Ace) was purchased from DS Pharma Biomedical Co., Ltd. (Japan). Human IgG (I4506), anti human IgG (I3382), and anti protein A (P3775) were obtained from Sigma Aldrich, Co., (USA). Rabbit anti SPEC (streptococcal pyrogenic exotoxin C) (R5V153-754) and sheep anti staphylococcal alpha hemolysin (S5V156-754) were purchased from Meridian Life Science, (USA). Rabbit polyclonal to streptococcal pyrogenic exotoxin B (anti SPEB) (ab53403) and mouse monoclonal to streptolysin (ab23501) were purchased from Abcam plc. (UK). Mouse anti SED (staphylococcus aureus enterotoxin D) (BM1327) was purchased from Acris Antibodies GmbH, (Germany). Bovine prothrombin (enzyme) (CP3049U) was purchased from Fitzgerald Industries International (USA). Mouse anti staphylococcus enterotoxin B, C2, D (anti SEB), (FU84002206) was purchased from Funakoshi Co., Ltd., (Japan). Non-homogenized milk was supplied by Kisuki Nyugyo Co. (Japan).

### 2.2. Fluidic Chip Structure and Fabrication

**Figure 3.**
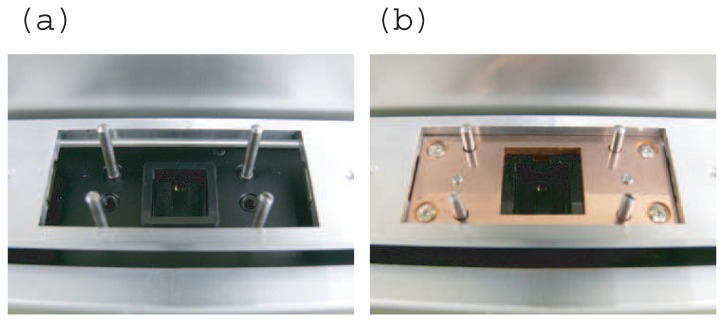
Close-up view of floating chip mounting system in (**a**) open state and (**b**) closed state. A black sensor chip in a black enclosure is located in the center of (a). The attitude of the sensor chip to the SPR instrument is adjusted by the leveling force of the float shown in (b).

Figure 1(a,b) shows an SPR instrument attached to a floating chip mounting system and an enlarged image showing an example of its use, respectively. Detailed photos of the floating chip mounting system in open and closed states are shown in Figure 3. The top and rear of the main components of the chip are shown on the left and right respectively in Figure 1(c). Figure 1(d) is a cross sectional drawing of the chip. The chip consists of 7 components as shown in Figure 1(e–k). The hatched areas in Figure 1(d) correspond to the legends in Figure 1(e–k).

Titanium was sputter-deposited on the injected-molded substrate (APEL™) to a depth of about 1.5 nm with SMH-2206RE sputter-deposition equipment (Ulvac Inc., Japan). Then gold was sputter-deposited to a depth of about 48.5 nm without breaking the vacuum. Gold/titanium film covered the whole of one surface of the substrate.

Two rectangular windows were opened in the adhesive film (GD-60-21S) using a mechanical cutting plotter (CG60ST Mimaki Engineering Co., Ltd., Japan) to form frames for the gel (Figure 1(i)). This adhesive film was attached to the non-sputtered surface of the substrate. Twenty-four substrates were set on the XY stage of a desktop robot (Shot Master 3, Musashi Engineering Inc., Japan) with the frame film side up.

An uncured solution of gel (IVS5022) was prepared according to the instruction manual (two liquids with the same weight were mixed and any air bubbles were removed by decompression) and placed in the syringe of a precision fluid dispenser (SMP-III, Musashi Engineering Inc., Japan). The solution was continuously dispensed onto the substrate inside the frame by drawing a spiral outward from the center of each window. Then the substrate was cured at 80 °C for 20 hours after a sufficient rest time until the surface of the uncured solution reached a steady state.

Ligands (antibody or enzyme) and blocking reagent were diluted with ion-exchange water and immobilized on the gold surface side of the gel-cured substrate using a spotter (Nano-Plotter 2.1, Gesim mbH, Germany) with a band array configuration. The concentration of all the ligands was 1 mg/mL. Each band was 0.2 mm × 0.44 mm in size and was arranged parallel to the band center at a distance of 0.25 mm within the SPR sensing area. There were 19 bands; 9 ligand bands and 10 reference bands (blocking reagent). The reference bands were arranged on either side of the ligand bands. After 30 minutes of incubation, the gold surface including the spot area was washed in running water to remove excessive sediment from the gold surface, and then exposed in blocking solution (2% blocking reagent diluted with water) for 20 minutes to reduce the contribution of the non-specific binding effect. The gold surface was again washed in running water and then air-dried.

Two-sided adhesive film (TL470S, 0.075 mm thick) was cut using a mechanical cutting plotter (CG60ST Mimaki Engineering Co., Ltd., Japan) to provide a flow channel (Figure 1(g)).

A capillary pump (Figure 1(f)) was fabricated with a CO_2_ laser cutting-machine (VL 200, Universal Laser Systems Inc, USA) from a large black acrylic resin base plate (3 mm thick). The integrated capillary tubes and inlet hole (3 mm diameter) were opened along the thickness direction and the lengths of all the capillary tubes and the inlet hole were the same as the thickness of the base plate. It took a few minutes to complete the capillary pump. The capillary radius was 0.136 mm. The distance between adjacent capillary centers was 0.36 mm. The number of capillaries and the total capacity of the integrated capillaries were 816 and 0.151 mL, respectively.

The capillary pump, flow channel film and SPR substrate with ligands and gel were assembled using a custom-made alignment jig. This chip-core size was 16 mm (W) × 16 mm (D) × 3 mm (H).

The chip-core was set in the outer enclosure (Figure 1(k)) and then the inner enclosure (Figure 1(e)) was inserted from the top until the four side locks were engaged. The chip-core was fixed tightly between the outer and inner enclosures and there was no backlash (see Figure 1(d)). The final product size and weight were 20 mm (W) × 20 mm (D) × 8 mm (H) and 2.3 g, respectively.

A gel protective cover (not shown) was attached to the bottom of the final product, which was packaged in an aluminum sealing bag with a desiccating agent and stored in a refrigerator until the measurement was performed.

### 2.3. Floating Chip Mounting System

**Figure 4.**
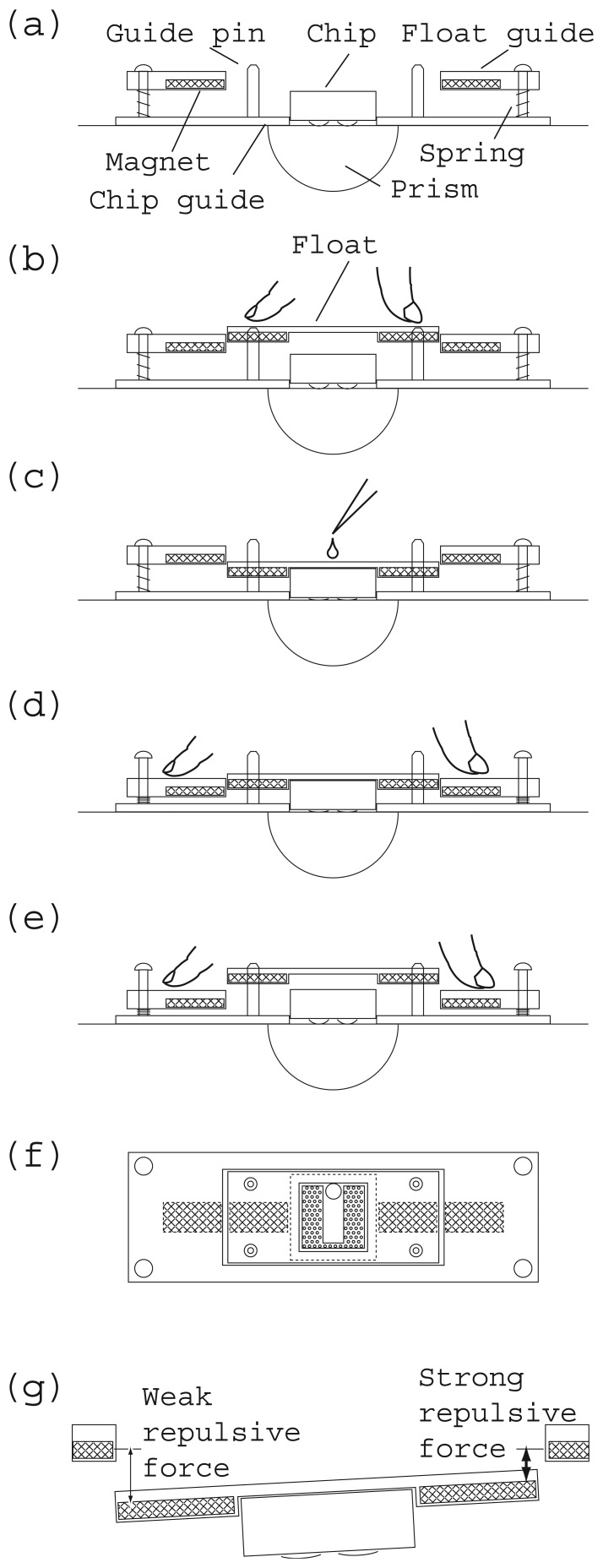
Operation of floating chip mounting system, chip is attached to prism (**a**), float is attached and pressed down (**b**), test solution is introduced and SPR measurement is carried out (**c**), float guide is pressed (**d**) and float returns to initial position (**e**). Top view of floating chip mounting system and chip (**f**). Autonomous attitude control mechanism (**g**).

Figure 4 is a schematic illustration of the operating principle of the floating chip mounting system. Figure 4(a–e) and (f) are sectional and top views, respectively. The hatched areas indicate magnets.

A measurement chip is installed on the prism of the SPR instrument and its position is adjusted using the instruments chip guide (Figure 4(a)). A float is positioned over the chip by passing four guide pins through corresponding holes (Figure 4(b)). The float is floating above the chip because the two float magnets experience repulsive forces from the closest float guide magnet and the resultant force is exerted upwards.

Next the float is pushed downwards manually against the upward force until the float passes through the rectangular hole in the float guide (Figure 4(c)). This time, the chip receives downward force from the float, which receives repulsive downward force from the float guide. Then the refractive matching gel couples the chip and prism optically. In this state an immunoassay is carried out by introducing a sample liquid from a pipette (Figure 1(b), Figure 4(c)).

After the measurement, the float guide is pushed downwards manually against the compression coiled springs until it reaches the bottom (Figure 4(d)). At this stage, the float is higher than the float guide.

The float then springs back to its initial position because the float experiences upward force from the float guide (Figure 4(e)). After that, the float and the chip are removed and these operations are repeated with the next sample.

### 2.4. Measurement

Reflection images were recorded by the CCD camera of the SPR instrument and transferred to the PC at three speeds. The mounting mechanism was analyzed using high speed recording at 0.1 s intervals (50 frame/s, 5 accumulations, 7.0 ms exposure time). Stability was studied using low speed recording at 25 s intervals (2 frame/s, 50 accumulations, 7.0 ms exposure time). An immunoassay was performed using medium speed recording at 1 s intervals (50 frames/s, 50 accumulations, 7.0 ms exposure time). The immunoassay was carried out with two-step direct injections without remounting the chip. The first injection was a 4 *μ*L reference solution (10% of blocking reagent). The second injection was an actual IgG spiked sample. The first injection of 4 *μ*L was performed and the solution remained in the center flow channel. We waited for 300 s and then injected the second sample. We considered this waiting time sufficient for the activation of the dried antibodies, enzyme and blocking reagent.

Once all the CCD images have been recorded on PC, we can later generate sensorgrams at any position in the sensing region. The immobilized ligand (antibody, enzyme) positions can be identified exactly from the SPR dip angle on the ligands by comparison with that on the gold surface. Therefore, in these measurements it was not necessary to remount the same chip at exactly the same position and/or to adjust precisely the horizontal position in relation to the SPR instrument.

## 3. Results and Discussion

### 3.1. Refractive Index Matching Gel

Cured refractive index matching gel was formed in each window of the rear of the SPR substrate as shown on the right in Figure 1(c). The gel covered the entire area inside the window. The gel was shaped like water droplets that rose in the rectangular frames. The gel was thickest at the center of the window. The gel adhered strongly to the SPR substrate and was not torn off even after repeatedly attaching the substrate to and removing it from the prism surface.

**Figure 5.**
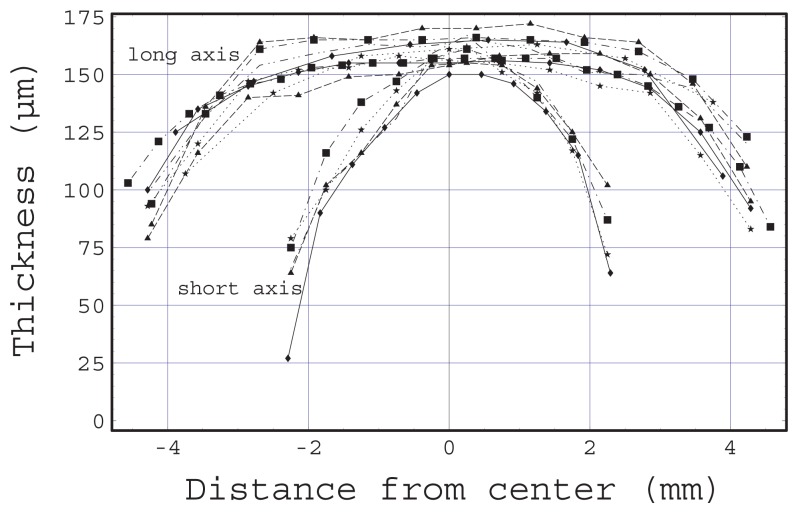
Thickness of gel along long axes and short axes as a function of distance from center. Several measurement results are superimposed.

Figure 5 shows the measured gel thickness profile as a function of the distance from the center. Many cured gels were cut with a sharp blade at the center of the window along the long and short axes shown in Figure 1(j) as L and S, respectively. The thickness was measured using microscope observations of a cross-section of each cut gel surface. There are two groups of lines, namely the thickness profile along the long axis and that along the short axis. The thickness profile along the long axis has a long plateau at the maximum thickness. However there were no plateaus in the thickness profile along the short axis. These thickness profiles are advantageous with regard to optical coupling to a flat prism surface in terms of the incursion of air bubbles. This is because when the gel comes in contact with the prism surface, the first contact occurs at the plateau and the contact areas then gradually expand to both sides of the plateau.

**Figure 6.**
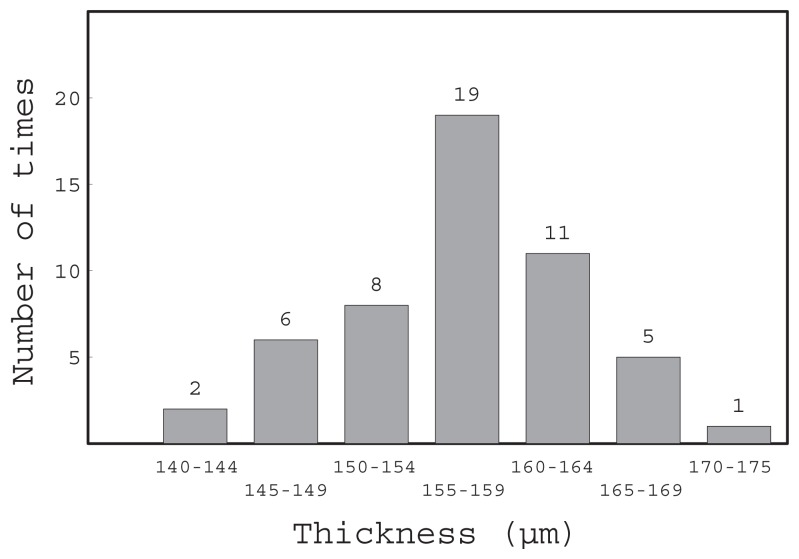
Frequency distribution of center thickness of gel.

Figure 6 shows the frequency distribution of the thickness of the center of the gels. We measured the thickness of the center of a total of 52 cured gels. The horizontal axis is the thickness range.

The gel has the same refractive index (nd = 1.51) as the glass prism. Therefore, the theoretical SPR dip position calculated from the light propagation in the planar metal/dielectric multiple layer based on electromagnetic theory is unchanged. However, in an actual instrument, the insertion of the gel meant that the total internal reflection point and the position at which the reflect light illuminated the CCD were displaced by x and y respectively, as shown in Figure 2(f). The displacement y on the CCD induces the apparent movement of the SPR dip position. These displacements x and y are proportional to the gel thickness *d* and are a function of incident angle *θ*, as expressed below by Equations (1) and (2), respectively.

(1) x=dtanθ.

(2) y=d/cosθ.

The thinner gel performs much as expected, because the SPR instrument is designed to use matching liquid whose thickness is negligible. The target thickness for the mounted state is about 0.1 mm, which exceeds the thickness of the frame film (0.08 mm, Figure 1(i)) and is less than that of the gel-center in the free state (0.17 mm). The movement in the SPR dip position is about 4% when we insert a 0.1 mm thick gel in our instrument. However, because the amount of displacement is the same over the entire sensing area, it has no influence on the reference-subtracted sensorgrams.

The average, maximum and minimum thicknesses of the gel at the center were 0.157, 0.172 and 0.143 mm, respectively. The fabrication error of the gel thickness was within ±0.015 mm. The misalignment of the SPR sensing area was calculated from this gel thickness error as ±0.04 mm. This value was in an acceptable range in relation to the flow channel width of 0.9 mm.

We designed the system so that the two gels were located on either side of the focus line of the cylindrical prism. The refractive index matching gel is not required to cover the entire rear surface of the substrate, but it is sufficient if it covers only the light path. Figure 1(d) shows this condition. The incident light passes through the left gel and substrate and is then reflected at the gold film that constitutes the bottom of the flow channel. It then passes through the substrate and right gel in sequence. This twin gel system has advantages with regard to attitude stability and in reducing the amount of gel. In addition, reducing the contact area enables us to mount the chip with little force. This allows us to downsize the mounting mechanism and to use a polymer substrate.

### 3.2. Autonomous Chip Attitude Control

Total internal reflection at a chip surface on which gold film has been deposited is a prerequisite of SPR measurement. A wedge shaped incident light and a wedge shaped reflected light were designed to be located over the critical angle even if the sample was air or water (Figure 2(b)). A CCD camera detects wedge shaped reflected light with a 480 × 640 pixel resolution. If the chip is mounted in an appropriate position without tilt, the CCD can detect all the wedge shaped reflected light (Figure 2(d)). Since the wedge shaped light is under a total internal reflection condition, if the sample on the gold has a resonance angle in the wedge shaped light, we can observe an SPR dip (Figure 2(e)). However, if the chip is mounted with tilt, the CCD detects none or only a part of the wedge shaped reflected light (Figure 2(c)). Then the total intensity of the CCD, namely the sum of the individual pixel outputs, is a reasonable factor for evaluating the position and tilt of the chip on the prism.

**Figure 7.**
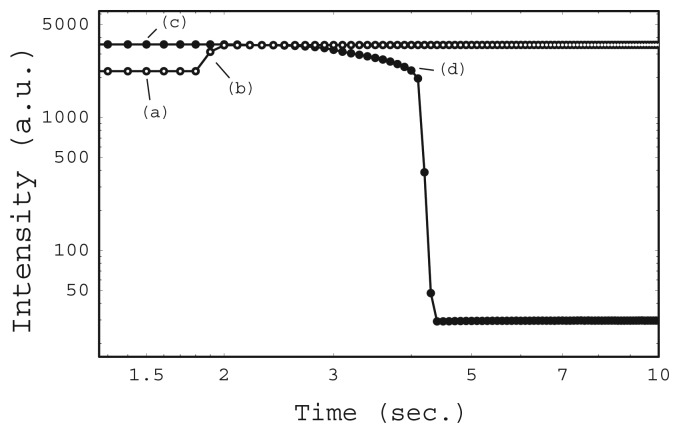
Total intensity of CCD in SPR instrument as a function of mounting time, repulsive magnetic force driven (open circles) and attractive magnetic force driven (filled circles).

Figure 7 shows the total intensity as a function of the mounting time. Two mounting systems are compared in the figure. One system is driven by the repulsive force of a magnet (open circles). The other is driven by the attractive force of a magnet (filled circles). The measurement interval was 0.1 s for each experiment. The lines connecting the circles are guides for the eyes.

The attractive force experiment was carried out using the mounting system shown in Figure 1(a) but no float guide, no springs and no pins located inside the springs. We used the array of identical ligand bands immobilized on a chip described in the experimental section. The chip guide was covered with an SPCC plate with the same shape as the chip guide and it was fixed tightly to the instrument with screws. Then attractive force was generated between the SPR instrument and the float.

**Figure 8.**
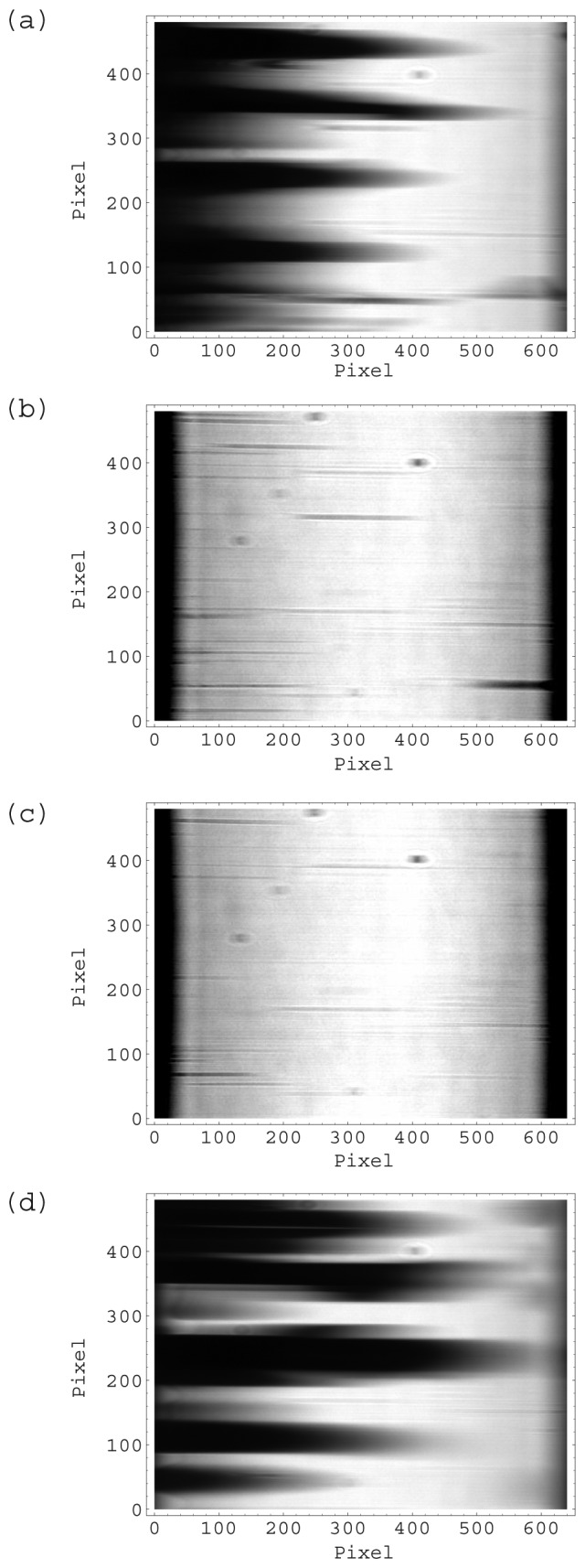
CCD output of SPR instrument at typical points indicated in Figure 7 (**a**–**d**). The horizontal axis is the angle of the reflected light. The vertical axis is the sensing position along a focal line (See Figure 2(b)).

In a repulsive force experiment, the chip was attached to the prism surface by inserting it in the chip guide without any special adjustment. At this initial stage, the chip was not mounted correctly as shown in Figure 8(a). Most of the CCD was occupied by dark areas, where no reflection was detected. The total intensity at this time is shown in Figure 7(a). Then the float was pushed by hand and the chip began to experience downward force from the float after 0.3 s from position (a). The total intensity increased immediately (Figure 7(b)), because the wedge shaped reflected light illuminated the entire CCD. Although many thin lines remain in image (b), these lines had disappeared one minute later.

In the attractive force experiment, the chip was carefully attached to the prism surface so that the wedge shaped reflected light illuminated the entire CCD area as the initial state. The CCD image at this position is shown in Figure 8(c). The total intensity (Figure 7(c)) was almost the same as that of the final state of the repulsive force experiment. The float was carefully attached to the chip while keeping the float horizontal. However, the total intensity decreased gradually at the beginning, and then decreased sharply after 4 s (Figures 7(d) and 8(d)). The wedge shaped reflected light could not illuminate the CCD completely when the chip was tilted. The float has 4 holes for guide pins. The difference between the diameter of the guide-pin holes and that of the guide pin was 0.5 mm to realize smooth movement of the float. The tilt angle was small as it was limited under this geometrical condition.

Even if the initial tilt angle of the float is extremely small, the attractive force generated between the left magnet and the SPCC plate differs from that between the right magnet and the SPCC plate. As the force difference increases, there is an increased tendency to tilt, because the attractive force becomes stronger as the distance decreases. Figure 5 reveals the possibility of there being a difference of up to 0.035 mm between the thicknesses of the left and right gels. It is difficult to realize gels with exactly the same thickness with the dispensing method.

However, the repulsive force driven mounter is stable even if there is initial tilt due to the different thicknesses of the gels. The float guide is parallel to the prism surface. When the float tilts with its left side down, the right magnet of the float and the right magnet of the float guide approach each other, and the left pair move away. The repulsive force becomes stronger as the distance decreases. The right magnet of the float experiences stronger downward force than the left magnet. Then the float works to eliminate the initial tilt. Thus the float achieves an autonomous attitude control function that keeps the chip parallel in relation to the prism surface of the SPR instrument (Figure 4(g)).

Another advantage of this floating chip mounting system is that the chip can be easily removed. When the float guide is pushed to the bottom by hand, the float returns to its initial floating position for the next chip. This function allows rapid chip exchange, thus satisfying the demand for a large number of measurements.

### 3.3. Stability Study for SPR Measurement

**Figure 9.**
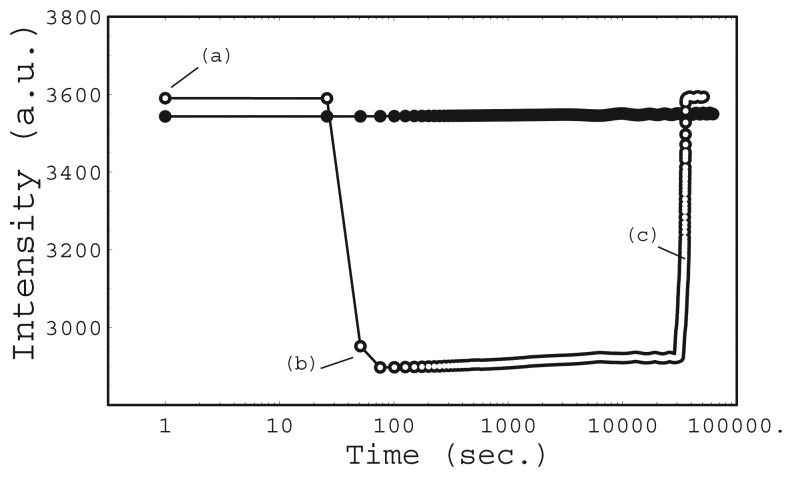
Stability experiments of repulsive force driven mounting system. Total intensity of CCD in SPR instrument as a function of mounting time, with no water introduced into chip (filled circles) and with water introduced (open circles).

Figure 9 shows the result of a long-term stability experiment on the floating chip mounting system. Open circles show the experimental results after 14.2 hours. 40 *μ*L of water was introduced into the inlet of the chip from a pipette 30 s after the measurement started. Another 40 *μ*L of water was introduced when the inlet hole was empty. Filled circles show the experimental result after 17.2 hours but without the introduction of water. The lines connecting the circles are guides for the eyes. The experiment without water shows that the total intensity maintained an almost constant value. The difference between initial and final intensities divided by the initial intensity was 1.7 × 10^−3^. The difference between the initial and final intensities of the two experiments was the result of using another chip.

**Figure 10.**
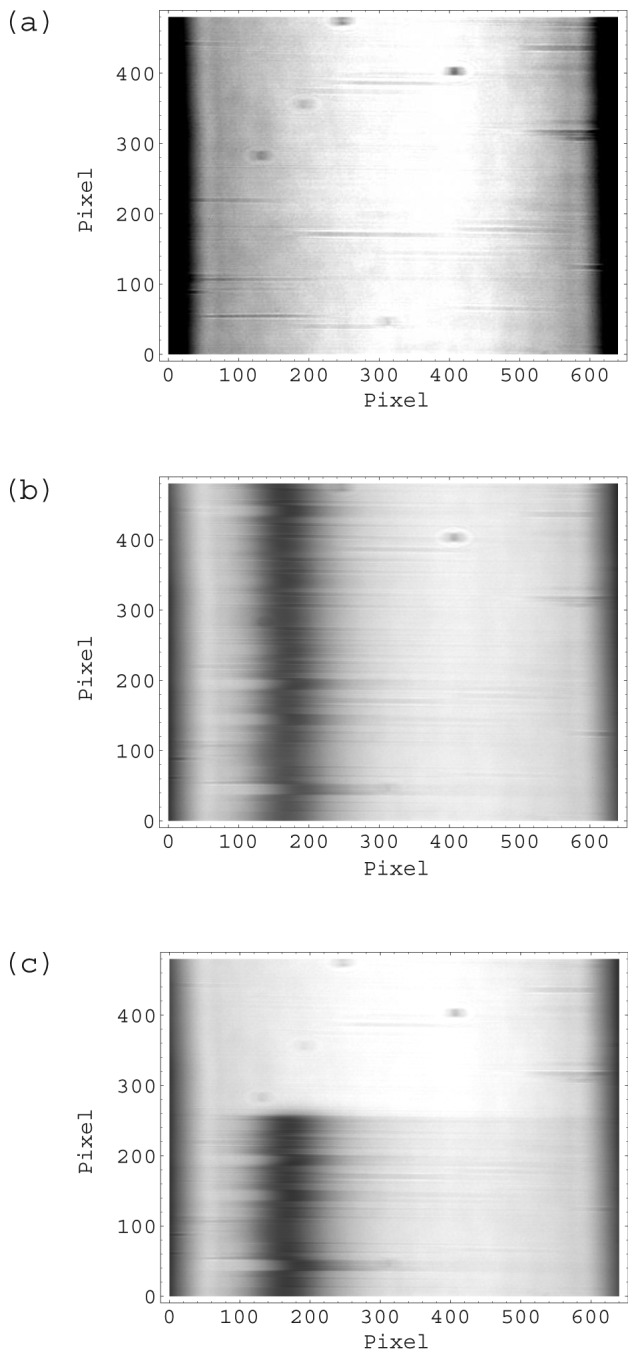
CCD output of SPR instrument at typical points indicated in Figure 9 (**a**–**c**). The horizontal axis is the angle of the reflected light. The vertical axis is the sensing position along a focal line (See Figure 2(b)).

The total intensity in the experiment in which water was introduced changed greatly. Some CCD images of typical points indicated in Figure 9 are shown in Figure 10. Figure 10(a) shows that the chip was mounted normally. The total intensity has decreased greatly in Figure 9(b). This is because the water flowed in the flow channel and there was surface plasmon resonance resulting in a decrease in total intensity. This SPR can be seen clearly in Figure 10(b). The dark vertical band is the SPR dip, namely the reflection minimum. In the dark band, 19 narrow horizontal bands can be seen. These narrow bands were immobilized antibodies, enzyme and blocking regents on the gold film as described in the experimental section. However, the total intensity was increased at the end of measurement in Figure 9(c). The water in the flow cell evaporated and the sensor chip no longer met the SPR condition (Figure 10(c)).

**Figure 11.**
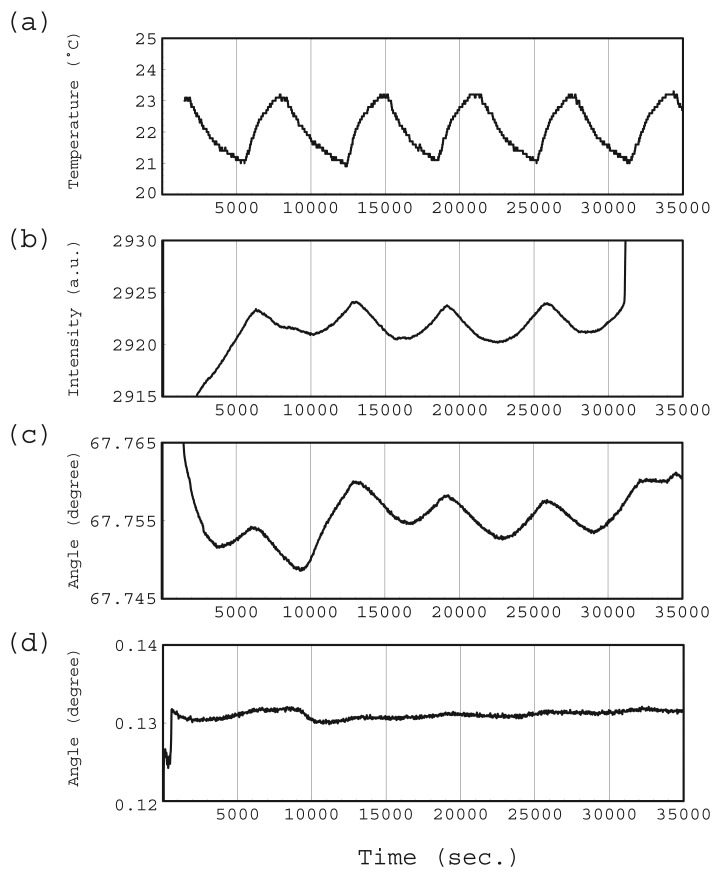
Drift measurements, laboratory temperature (**a**), intensity measured with SPR instrument (**b**), SPR angle at antibody (**c**), SPR angle where the reference angle was subtracted from the antibody angle (**d**).

We found small fluctuations in the total intensities in both measurements. Figure 11(b) is the result of re-plotting the same water introduction experiment with a linear time scale and expanded intensity. The laboratory temperature measured for the same period is shown in Figure 11(a). Both graphs show periodic waves with the same cycle length but a different phase shift except at the start of the experiment and after 31,000 s, when the water starts to evaporate. The temperature wave is caused by the air-conditioning cycle.

Figure 11(c) is the SPR angle where the reflection intensity is minimum. This is observed at an immobilized antibody S5V156-754 as one example. The SPR angle also changed with the temperature.

The differencing operation, subtraction the reference signal from the target antibody signal, was very effective in removing nonspecific antigen-antibody binding signals from a real sample, which includes many kinds of foreign substances. This subtraction is also effective in reducing thermal drift. Figure 11(d) shows an SPR angle where the reference angle has been subtracted from the target antibody angle. The reference angle is the average value measured at immobilized reference reagent (Block Ace) areas located on both sides of the target antibody. The full-scale range of the angle axis is 0.02 degrees in both Figure 11(c) and Figure 11(d). The SPR angle fluctuation caused by thermal drift was reduced to one-fifth its original value.

Although errors caused by thermal drift can be ignored in an actual SPR immunoassay because a typical antigen-antibody reaction is completed within several minutes if the antigen concentration is sufficient, this SPR instrument mounting system has a beneficial effect on long-term monitoring.

### 3.4. SPR Immunoassay Demonstration

**Figure 12.**
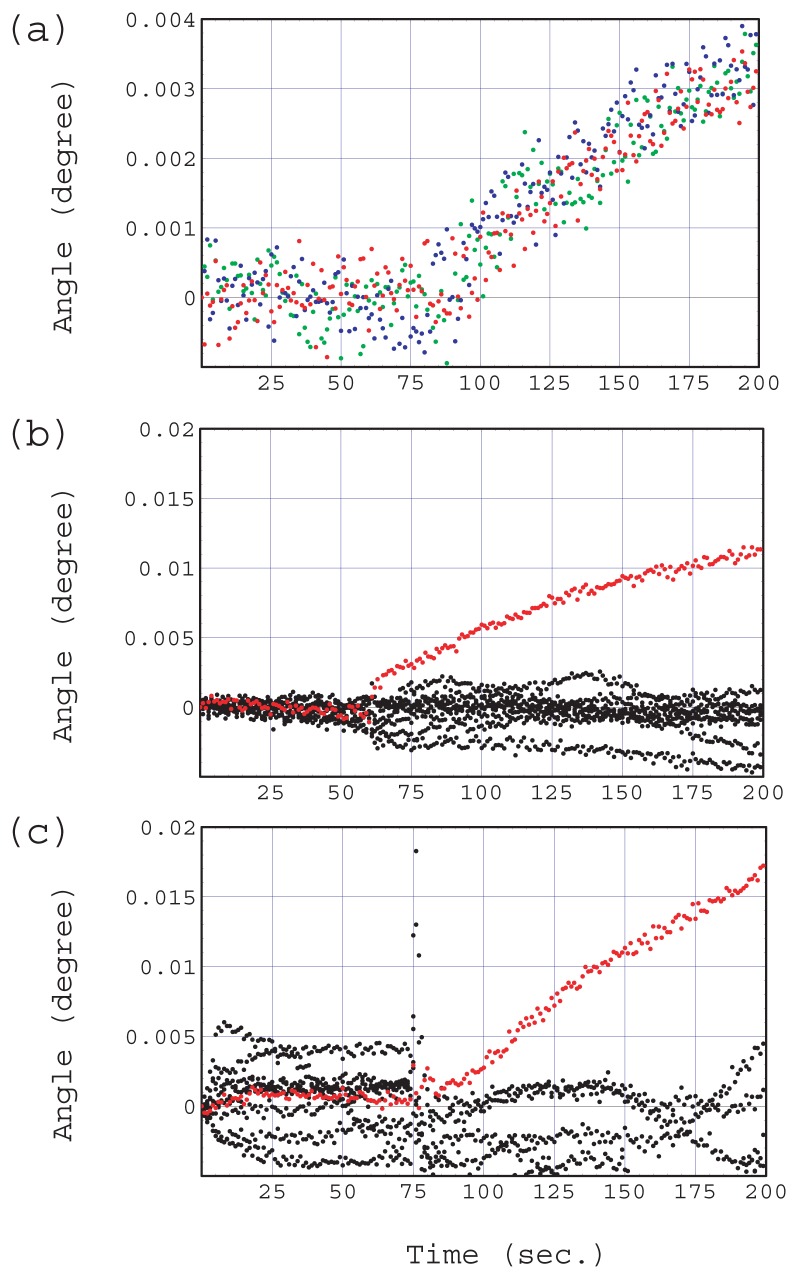
Typical immunoassay sensorgrams. The sensor chip was mounted using refractive index matching oil and sample solution is 1 *μ*g/mL human IgG in the 10% blocking reagent. Three antibody bands responses are plotted in different colors (**a**). The sensor chip was mounted using the developed system and the sample solution was 5 *μ*g/mL human IgG in the 10% blocking reagent (**b**). The sample solution was 5 *μ*g/mL human IgG in a real sample (non-homogenized milk) and the other condition was the same as (b), (**c**).

Figure 12 shows typical SPR immunoassay sensorgrams measured with different chips. All the sensorgrams were obtained by subtracting an average reference sensorgram from that of an antibody. Here the average reference sensorgram is the average of reference sensorgrams measured on blocking reagent bands located on both sides of the target antibody. Figure 12(a) shows sensorgrams obtained in a comparison experiment to evaluate the developed mounting system. The sensor chip was fabricated in the same manner except that there were no gel sheets on the reverse side. The substrate was glass (BK7). Three antibody (anti human-IgG) bands and four blocking reagent (Block Ace) bands were immobilized with a band array configuration in an alternating arrangement. Refractive index matching oil was used for the optical coupling to the prism. A sample solution of 1 *μ*g/mL human-IgG in a reference solution (10% blocking reagent) was injected after 300 s activation with the reference solution. Three sensorgrams on human-IgG bands were plotted in different colors. The three sensorgrams showed similar responses. A clear elevation caused by an antigen-antibody binding reaction was observed at 75 s, the time at which the sample was injected. The slope of a tangential line at the injection time was about 4.0×10^−5^ degrees per second.

SPR immunoassay sensorgrams measured with the developed chip and mounting system are shown in Figure 12(b). The sample solution was 5 *μ*g/mL human-IgG in reference solution. The red dotted line shows the response of anti human-IgG and the black dotted lines are the responses of the other eight ligands. Only the specific binding of the antigen-antibody reaction was observed as an elevation in the sensorgram. The slope of the tangential line was about 2.0×10^−4^ degrees per second. Figure 12(c) shows the result of the same immunoassay experiment using 5 *μ*g/mL human-IgG but in non-homogenized milk, namely a model real sample, rather than the reference solution. Only the specific binding of the antigen-antibody reaction (red dotted line) was observed in a similar way. The slope was about 1.8 × 10^−4^ degrees per second. Non-homogenized milk contains many foreign substances such as fat globules (0.1–15 *μ*m in diameter) and its refractive index is very different from that of the reference solution. It is considered that these factors increase the instability in sensorgrams (c).

We believe that the usefulness of our sensor chip and its mounting system for on-site immunoassay is confirmed by the clear detection of specific of antigen-antibody binding and the reasonable relationship between the antigen concentration and the sensorgram slope.

## 4. Conclusions

We have developed a chip mounting system that offers an easy and simple operation for a portable SPR measurement instrument. Two sheets of refractive index matching gel formed on the rear of the SPR chip were used to realize optical coupling with the prism of the SPR instrument. The chip mounting mechanism driven by the repulsive force of permanent magnets controls the chip attitude automatically and thus keep the chip parallel to the prism surface. The fabrication error regarding the thickness of the two gel sheets was easily overcome, because the chip attitude control mechanism absorbed this error. The chip mounting state was very stable in a load test lasting several hours. We confirmed the usefulness of our developed system by performing an immunoassay experiment on a real sample. The fact that the gels are deployed on the side of the chip allows the advantage of the self cleaning of the prism surface. Even if small unwanted particles accidentally intrude between the prism and the chip during the chip exchange procedure, we can determine their existence from a CCD image obtained before measurement. After removing this chip from the instrument, we found that the most of the particles had adhered to the gels. These particles were easily removed without the gel breaking by using commercially available adhesive tape because these tapes adhere to the particles but not to the silicone gel surface. Measurement can then be restarted using the same chip with a clean prism surface.

We expect this mounting system to be applied to both SPR instruments and in many fields where reversible coupling technology is required.
